# To explore the performance of ultrasound elastography in staging diabetic kidney disease: a systematic review and meta-analysis

**DOI:** 10.1038/s41598-026-39278-w

**Published:** 2026-02-06

**Authors:** Alisa Mohebbi, Saeed Mohammadzadeh, Fatemeh Asli, Faeze Salahshour, Afshin Mohammadi

**Affiliations:** 1 Universal Scientific Education and Research Network (USERN), Tehran, Iran; 2 School of Medicine, Tehran University of Medical Sciences, Tehran, Iran; 3 Department of Radiology, Tehran University of Medical Sciences, Imam Khomeini Hospital, Tehran, Iran; 4 Department of Radiology, Faculty of Medicine, Urmia University of Medical Science, Urmia, Iran

**Keywords:** Ultrasound elastography (USE), Diabetes mellitus (DM), Diabetic kidney disease (DKD), Shear wave elastography (SWE), Diagnostic test accuracy (DTA), Diseases, Endocrinology, Health care, Medical research

## Abstract

To assess the diagnostic performance of different quantitative ultrasound elastography (USE) parameters in differentiating five stages of diabetic kidney disease (DKD) from each other as well as from healthy participants and type 2 diabetic patients without nephropathy. The protocol was pre-registered at https://osf.io/dfu8b/. Using PubMed, Web of Science, Embase, and Cochrane Library, we found studies up to October 10, 2023. Risk of bias was assessed using Quality Assessment of Diagnostic Accuracy Studies-2 (QUADAS-2). The USE parameter of cortical stiffness was compared in an inter-stage manner based on mean differences, standardized mean differences, and ratios of means. In addition, cortical stiffness was evaluated for its diagnostic accuracy in staging DKD. Sensitivity and publication bias analyses were also conducted. Certainty of evidence was assessed using Grading of Recommendations, Assessment, Development, and Evaluation (GRADE). Eighteen studies were included with 2814 participants. The mean stiffness value consistently increased with the progression of the disease. Differentiation of five stages of DKD from two non-DKD groups, respectively, showed sensitivity, specificity, positive likelihood ratio, negative likelihood ratio, diagnostic odds ratio, and area under curve of 79%, 83%, 4.70, 0.25, 18.68, and 0.88. These values for differentiation of stages 3 to 5 of DKD from the other four milder groups were 80%, 79%, 3.85, 0.25, 15.53, and 0.87, respectively. USE shows promise as a noninvasive, simple, and quantitative diagnostic tool for staging DKD, especially in its early stages. With standardized protocols, its application could benefit patient management by enhancing the accuracy of DKD staging.

**Supplementary Information:**

The online version contains supplementary material available at 10.1038/s41598-026-39278-w.

## Introduction

Diabetic kidney disease (DKD), the leading cause of renal failure worldwide, is regarded as a dominant predictor of mortality in diabetic patients^1^. Given the elevated prevalence and increasing incidence of diabetes, a large number of people are pursuing healthcare for diabetes-related renal impairment, leading to a substantial increase in healthcare costs and lowering patients’ quality of life^2,3^. When initiated in the early stages, effective treatment can greatly decelerate the rate of disease progression; therefore, early detection of diabetes-induced nephropathy is critical in determining patients’ prognosis^4,5^.

DKD typically begins with nuanced histological changes influenced by intrinsic renal compensatory mechanisms that can only be identified through renal biopsy. Nevertheless, reliance on biopsy for early detection and staging is impractical and frequently prohibitive due to its intrinsic limitations^6^. A kidney biopsy is an invasive technique that entails risks of complications such as bleeding and the possibility of acquiring an insufficient tissue sample. Moreover, strict clinical indications generally limit its application to instances of diagnostic uncertainty or rapidly progressing disease^6,7^. Current methods for DKD diagnosis mainly rely on measuring urinary albumin levels as the most clinically accepted and often reported criterion^8,9^. The application of albuminuria as a diagnostic indicator of diabetic nephropathy, however, has been challenged by several recent studies^5,8^. DKD can develop without pre-existing albuminuria and a considerable proportion of patients with diabetes can develop advanced DKD with new-onset microalbuminuria without progressing to proteinuria^9^.

Ultrasound Elastography (USE), a novel non-invasive imaging modality, has particularly gained attention in the past decade for its ability to quantify tissue stiffness. This method has already demonstrated efficacy in various diseases, such as liver fibrosis or focal lesions, portal hypertension (via spleen stiffness), and thyroid, breast, or prostate lesions. In these clinical contexts, it is routinely used for non-invasive diagnosis, risk stratification, and treatment monitoring^10,11^. In chronic kidney disease, USE measures the stiffness of the renal cortex, providing information about underlying tissue fibrosis and injury^12^. USE results have shown good correlation with renal function markers such as serum creatinine and estimated glomerular filtration rate^13^. USE has proven to be valuable in the study of DKD, the principal cause of chronic kidney disease, by facilitating the quantitative assessment of disease progression. The observed increase in tissue stiffness is predominantly attributed to progressive glomerulosclerosis and interstitial fibrosis^6,14^. Some studies provide evidence regarding the potential of elastography in detecting early changes even before the onset of diabetic nephropathy, emphasizing its possible role in detecting even the slightest mechanical tissue changes in the course of the disease^7^. Keeping that in mind and considering the cost-effectiveness and non-invasiveness of elastography, this technique may be a valuable clinical tool in the diagnosis, staging, and follow-up of DKD^7,12^.

Several published original studies are available, investigating the efficacy of different elastography parameters in DKD diagnosis and staging. However, no systematic review with meta-analysis has been published so far to compare these parameters. Therefore, we conducted this study to investigate how these parameters may potentially differentiate DKD stages from each other and from diabetic patients without DKD or normal healthy participants.

## Materials and methods

This systematic review and meta-analysis was pre-registered on the Open Science Framework (OSF) at https://osf.io/dfu8b/ (see Appendix A). The methodology was developed in accordance with the Cochrane Handbook for Systematic Reviews of Diagnostic Test Accuracy^15^. Preferred Reporting Items for a Systematic Review and Meta-analysis of Diagnostic Test Accuracy (PRISMA-DTA) and Search (PRISMA-S) were used for reporting.

### Search strategy

Two reviewers independently designed search syntaxes. For the search strategy, EMTREE keywords and MeSH keywords were used. The two strategies were discussed, and a merged strategy was developed. A third reviewer was involved in discussions that failed to reach a conclusion. Search syntax for PubMed, Web of Science, Embase, and Cochrane Library was implemented without language restrictions on October 10, 2023 (Appendix B). Additionally, published conference abstracts were included as recommended by the Cochrane Handbook for Systematic Reviews of Diagnostic Test Accuracy^15^. We also reviewed the reference lists of relevant papers to identify any missing publications. All retrieved records were imported into EndNote software, and duplicate records were removed.

### Eligibility criteria & study selection

Cohort, cross-sectional, and case-control studies involving at least five individuals with type 2 diabetes mellitus (DM) who underwent renal elastography examination were considered for inclusion. Crucially, studies were only deemed eligible if they evaluated the performance of USE in assessing the stage of DKD, using albuminuria categories as the clinical reference standard for staging. The exclusion criteria comprised non-research article types such as reviews, case reports, editorials, and commentaries. When multiple publications utilized the same dataset, the study with the most comprehensive data was included for evaluation. If a study’s dataset was a subset of another study’s dataset, it was considered less comprehensive. To avoid including duplicate reports, the authors’ names of the studies that were included were cross-checked. Two reviewers conducted an independent screening of the search results, excluding studies that were not relevant. Any discrepancies in the included studies were resolved through discussions between the reviewers. A third reviewer adjudicated any disagreements that persisted after discussion.

### Risk of bias assessment

Two reviewers independently assessed the risk of bias using the Quality Assessment of Diagnostic Accuracy Studies-2 (QUADAS-2) tool in four domains of selection, index test, reference test, and flow and timing. Any unresolved disagreements between the evaluators were referred to a third reviewer.

### Data extraction

Two reviewers independently performed data extraction. Data entry was carried out using an Excel sheet, and the files were cross-checked to identify any inconsistencies. In the event of unresolved disagreements, the third reviewer was consulted for resolution. The third reviewer resolved all discrepancies by reviewing the source material, the initial assessments, and the arguments of the other two reviewers, providing a final, binding decision.

According to Kidney Disease Improving Global Outcomes (KDIGO) criteria^16^, urine albumin-to-creatinine ratios (ACR) < 30 were considered normoalbuminuria, 30–300 as microalbuminuria, and > 300 as macroalbuminuria for reference testing. DKD is staged based on declining kidney function, measured by the estimated glomerular filtration rate (eGFR). Stage 1 indicates kidney damage with normal/high eGFR (≥ 90), Stage 2 mildly decreased eGFR (60–89), Stage 3 moderately decreased (30–59), Stage 4 severely decreased (15-29), and Stage 5 kidney failure (< 15). Also, based on literature^6,17^, DKD stages were categorized according to standard albuminuria thresholds: normoalbuminuria for early-stage disease (Stages 1–2), microalbuminuria for stage 3, and macroalbuminuria for advanced disease (Stages 4–5). To facilitate grouping, patients with diabetes but without nephropathy are referred to as stage 0 and healthy controls as stage − 1 throughout this text. Given that USE data were reported interchangeably as stiffness (kPa) or velocity (m/s), we converted all velocity values to kPa to enable a unified meta-analysis. The conversion was performed using the established formula E = 3ρV², assuming a kidney density (ρ) of 1.05 g/cm³.

We extracted 2x2 contingency diagnostic data were available, focusing on three key diagnostic thresholds that compared lower stages of DKD against higher stages. These differentials were: (A) (−1 + 0) vs. (1 + 2 + 3 + 4 + 5); (B) (−1 + 0 + 1 + 2) vs. (3 + 4 + 5); and (C) (−1 + 0 + 1 + 2 + 3) vs. (4 + 5). True positive (TP) was defined as a patient with the higher stage of DKD correctly identified by USE. Conversely, true negative (TN) was a patient with the lower stage of DKD correctly identified by USE. An incorrect classification of a lower-stage patient as having the higher stage constituted a false positive (FP), while a higher-stage patient incorrectly classified as having the lower stage was deemed a false negative (FN).

### Data synthesis

Statistical analysis was conducted using STATA version 17.0, MedCalc version 20.0, and Psychometrica. Sensitivity analyses and publication bias tests were also conducted. In studies where the effect size and its confidence interval were reported in a stage-wise manner, we pooled them before incorporating them into the overall pooled analysis with other studies. A similar approach was made if the data for the right and left kidneys were reported separately without an overall value. This approach follows guidance from the Common Mistakes in Meta-Analysis book^19^.

To evaluate the change in cortical stiffness across disease progression, we performed a meta-analysis of quantitative outcomes and synthesized pooled cortical stiffness for each distinct DKD stage. We further assessed the quantitative differences between stages by pooling the mean differences (MD), standardized mean differences (SMD), or percentage change in cortical stiffness. These comparisons utilized a random-effects model to account for clinical and methodological heterogeneity. In instances where the original studies failed to report MD, SMD, or percentage change, these effect sizes were calculated utilizing the available data. For studies where 2x2 contingency data were available, we performed a diagnostic test accuracy meta-analysis using a random-effects bivariate model to pool key diagnostic performance metrics, including sensitivity and specificity, the area under the curve (AUC), positive likelihood ratio (PLR), negative likelihood ratio (NLR), and diagnostic odds ratio (DOR). The calculated effect sizes were subsequently incorporated into the meta-analysis. We also interpreted the magnitude of the AUC, likelihood ratios, SMD, and percentage values according to their guidelines^20–23^. For diagnostic accuracy meta-analysis, we extracted sensitivity, specificity, TP, FP, FN, and TN from each included study for each DKD stage comparison. Between-study heterogeneity in diagnostic accuracy was assessed through I². We also constructed ROC curves displaying the sensitivity-specificity coordinates from each study, which provide visual representation of heterogeneity and the variation in diagnostic accuracy across studies. Random-effects bivariate models were employed for all diagnostic accuracy meta-analyses. We applied the GRADE (Grading of Recommendations Assessment, Development and Evaluation) methodology to systematically assess the quality of evidence contributing to pooled diagnostic accuracy estimates. Based on systematic evaluation of these five GRADE domains, each diagnostic accuracy estimate was assigned an evidence quality rating: High certainty (further research is very unlikely to change our confidence in the estimate), Moderate certainty (further research is likely to have an important impact on our confidence in the estimate and may change the estimate), Low certainty (further research is very likely to have an important impact on our confidence in the estimate and is likely to change the estimate), or Very low certainty (we are very uncertain about the estimate). Also, when substantial heterogeneity was identified (I² > 50%), we investigated potential sources through subgroup analyses, meta-regression or sensitivity analysis where appropriate, rather than proceeding with pooled estimates that might not be clinically meaningful^24^. A p-value of < 0.05 was considered statistically significant.

## Results

### Search results and study characteristics

**Fig. 1:**
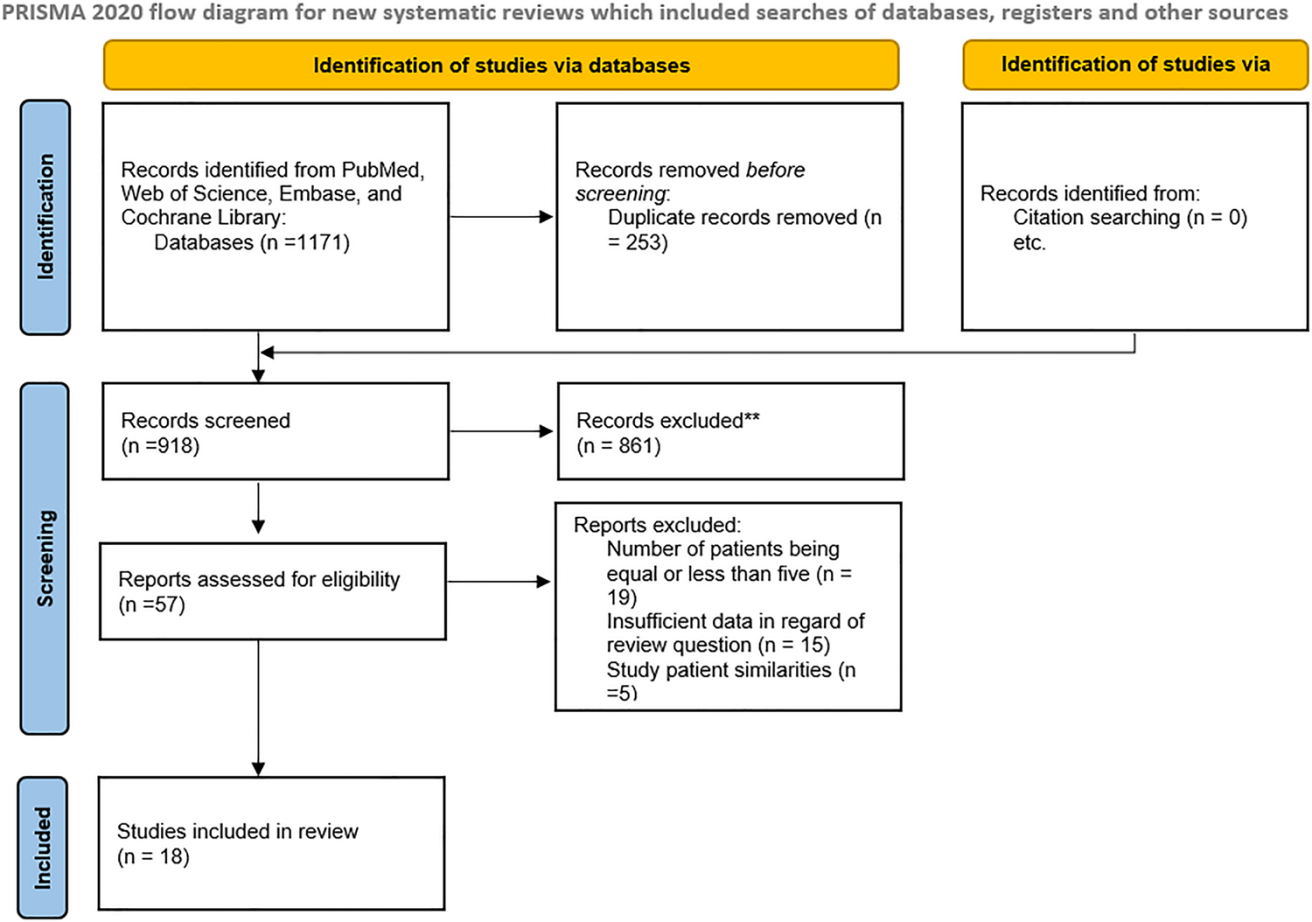
PRISMA flowchart. The leading causes for record exclusion** were: (**a**) studies that performed elastography examinations on the liver, spleen, or artery. (**b**) poor-quality conference abstracts (**c**) studies that assessed renal stiffness qualitatively.

A total of 18 studies were included, with 1803 type 2 DM and 955 healthy participants (Table 1). Figure 1 shows the PRISMA flowchart^4–7,10,11,14,17,18,25–33^. Of these studies, 13 had sample sizes of > 100 participants. The geographical distribution of studies shows a primary focus on East Asia (*n* = 8), followed by West Asia (*n* = 7), Europe (*n* = 2), and South Asia (*n* = 1).

**Table 1:** Included studies’ characteristics.

Name	Year	Publication type	Country	Elastography technique	Number of DMs	Number of healthy individuals	Number of normoalbuminuria	Number of Microalbuminuria	Number of Macroalbuminuria	Number of used radiologists with	years of experience	MHz
Yu et al. (2014)	2014	article	China	ARFI	120	30	29	19	18	2	15 years each	3.5–5.5.5.5
Goya et al. (2015)	2015	article	Turkey	ARFI	114	281	70	13	30	1	6 year	1–4
Yu et al. (2015)	2015	article	China	SWE	150	50	50	50	50	1	15 years	3.5–5.5.5.5
Hassan et al. (2016)	2016	article	Israel	ARFI	29	23	0	14	15	1	experienced	1–5
Lin et al. (2017)	2017	article	Taiwan	ARFI	223	91	0	147	80	1	>10 years	1–5
Bob et al. (2017)	2017	article	Romania	SWE	80	84	31	20	29	Not reported	Not reported	1–6
Koc et al. (2018)	2018	article	Turkey	Strain elastography	50	53	Not mentioned	Not mentioned	Not mentioned	1	5 years	1–5
Gungor et al. (2019)	2019	article	Turkey	SWE	75	None	Not mentioned	Not mentioned	Not mentioned	3	experienced	3.5
Koc et al. (2019)	2019	article	Turkey	SWE	120	22	48	26	46	Not reported	Not reported	1–5
Liu et al. (2019)	2019	article	China	ElastPQ	109	None	40	36	33	2	experienced	Not reported
Krishnan et al. (2020)	2020	article	India	ElastPQ	130	130	62	28	40	2	one having >5 years	1–5
Sumbul et al. (2020)	2020	article	Turkey	SWE	82	43	Not mentioned	Not mentioned	Not mentioned	Not reported	Not reported	1–5
Shi et al. (2020)	2020	article	China	SWE	93	31	51	12	20	1	Not reported	1–6
Sia et al. (2020)	2020	abstract	Singapore	SWE	58	None	6	34	18	Not reported	Not reported	2–5
Fang et al. (2021)	2021	article	China	SSI	187	56	75	41	71	1	5 year	2–5
Gunduz et al. (2021)	2021	article	Turkey	ARFI	40	17	18	Not mentioned	Not mentioned	1	12 years	Not reported
Yuksekkaya et al. (2022)	2022	article	Turkey	SWE	108	17	Not mentioned	Not mentioned	Not mentioned	1	15 years	1–6
Chirilia et al. (2022)	2022	abstract	Romania	SWE	35	27	0	35	0	Not reported	Not reported	Not reported

All included studies evaluated the diagnostic performance of quantitative ultrasound elastography using standardized diagnostic criteria for DKD classification. Every study classified diabetic kidney disease stages according to internationally recognized guidelines. These guidelines primarily used eGFR-based criteria in conjunction with albuminuria phenotyping as established by KDIGO and major nephrology organizations. While diagnostic criteria were uniform across studies, minor differences existed in imaging techniques employed. The majority of studies utilized shear wave elastography (SWE), while others employed other techniques. Minor variations in technical execution, such as probe frequency and region of interest (ROI) selection were noted.

### Risk of bias assessment

Appendix C provides a visual representation of the risk of bias assessment based on QUADAS-2 criteria. The primary reason for the selection bias observed in the included studies was the exclusion of patients with diabetes-related comorbidities, including hypertension, cirrhosis, etc. This phenomenon can lead to overfitting and upgrading biases. Since these comorbidities are common in clinical practice, studies should take this principle into account. The included studies exhibited minimal risk of reference standard bias and index test bias. The included studies also exhibited a brief time interval between the elastography assessment and the gold standard test, thus reducing the flow and timing bias.

### Group comparison based on cortical stiffness

A total of 2758 patients were included in the analysis, derived from 18 studies^4–7,10,11,14,17,18,25–33^. The pooled mean cortical stiffness values of patients were as follows: 11.7 kPa (CI = 8.4 to 15.0) for healthy control, 14.2 kPa (CI = 9.7 to 18.7) for DKD patients with normoalbuminuria, 17.0 kPa (CI = 13.5 to 20.5) for DKD patients with microalbuminuria, and 20.6 kPa (CI = 12.8 to 28.4) for DKD patients with macroalbuminuria. Data on stiffness in DM patients without DKD could not be pooled due to insufficient reported data. Appendix D presents forest plots for stiffness values across all participant groups in this review, ranging from healthy controls to DKD patients with macroalbuminuria.

**Fig. 2:**
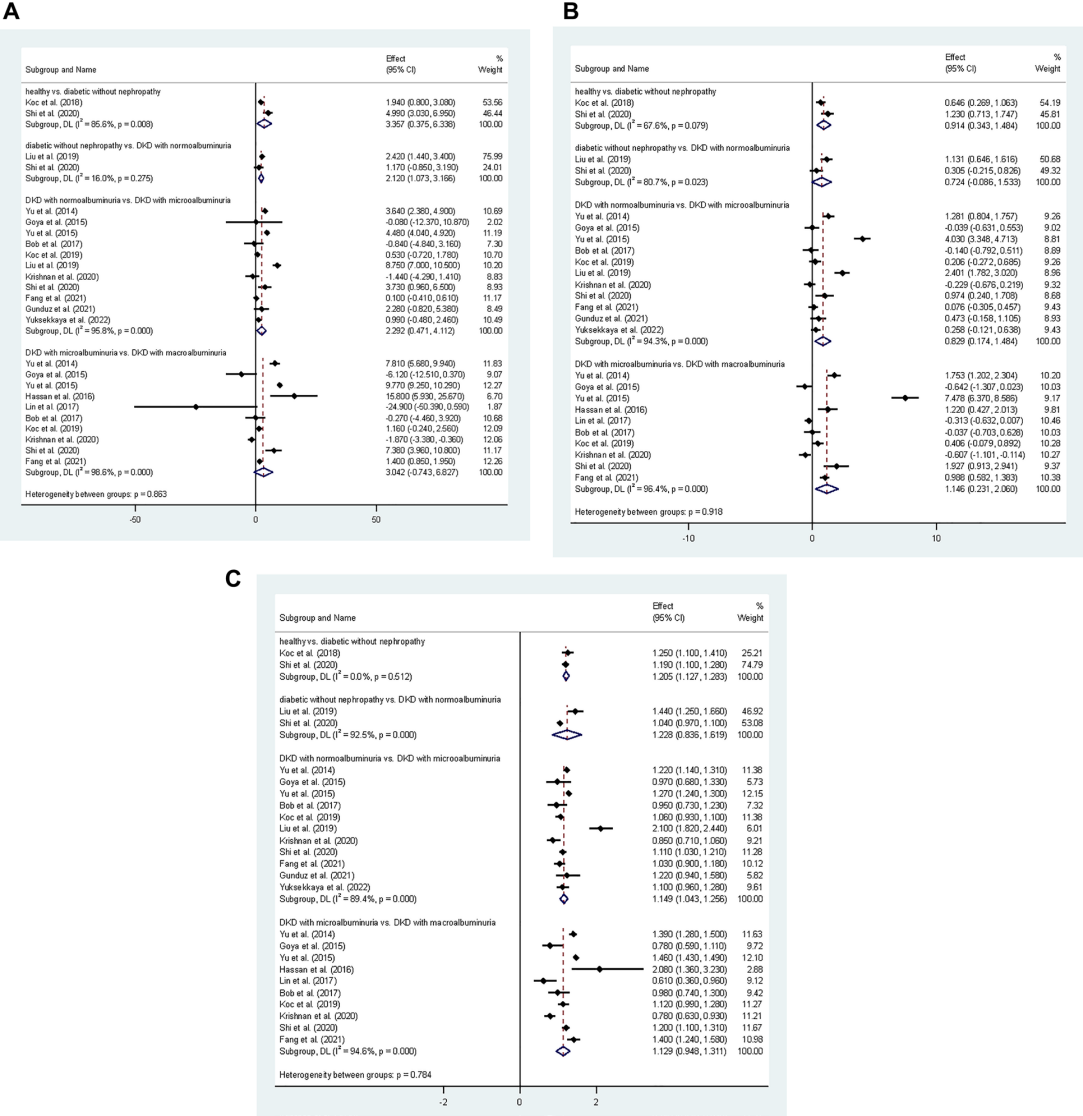
Results of the quantitative outcome meta-analysis using forest plots. Plot A illustrates the mean difference (MD) in cortical stiffness between different stages of Diabetic Kidney Disease (DKD). Plot B shows the standardized mean difference (SMD) in cortical stiffness for various DKD stage comparisons. Finally, Plot C represents the percentage change (presented as a ratio of mean stiffness values) in cortical stiffness across the progression of DKD stages. Diamond symbols denote pooled estimates, whereas horizontal lines represent 95% confidence intervals for individual investigations. Heterogeneity statistics (I²) are presented for each analysis.

To explore the differences in renal stiffness among groups, DM patients without DKD showed a + 20.5% (CI = + 12.7% to + 28.3%) increase in cortical stiffness compared to healthy control patients. Also, DKD patients with microalbuminuria had + 14.9% (CI = + 4.3% to + 25.6%) higher cortical stiffness values than those with normoalbuminuria. Finally, DKD patients with macroalbuminuria showed + 12.9% (CI = −5.2% to + 31.1%) higher cortical stiffness than those with microalbuminuria. Table 2; Fig. 2 represent the detailed comparison of reported data for cortical stiffness measured in kPa.

**Table 2:** Summarized results of the meta-analysis comparing the cortical stiffness of different stages of diabetic kidney disease (DKD). The pooled estimates for the change in stiffness across stages are presented using three key metrics of mean difference (MD), standardized mean difference (SMD), and the percentage change.

Parameter	MD	SMD	Percentage change
Cortical stiffness			
Healthy vs. Diabetic without nephropathy	3.357 (0.375 to 6.338)	0.914 (0.343 to 1.484)	+20.5% (+12.7% to +28.3%)
Diabetic without DKD vs. DKD with normoalbuminuria	2.120 (1.073 to 3.166)	0.724 (−0.086 to 1.533)	+22.8% (−16.4% to +61.9%)
DKD with normoalbuminuria vs. DKD with microalbuminuria	2.292 (0.471 to 4.112)	0.829 (0.174 to 1.484	+14.9% (+4.3% to +25.6%)
DKD with microalbuminuria vs. DKD with macroalbuminuria	3.042 (−0.743 to 6.827)	1.146 (0.231 to 2.060)	+12.9% (−5.2% to +31.1%)

### Diagnostic value of USE for differentiating (−1 + 0) vs. (1 + 2+3 + 4+5) stages

**Fig. 3:**
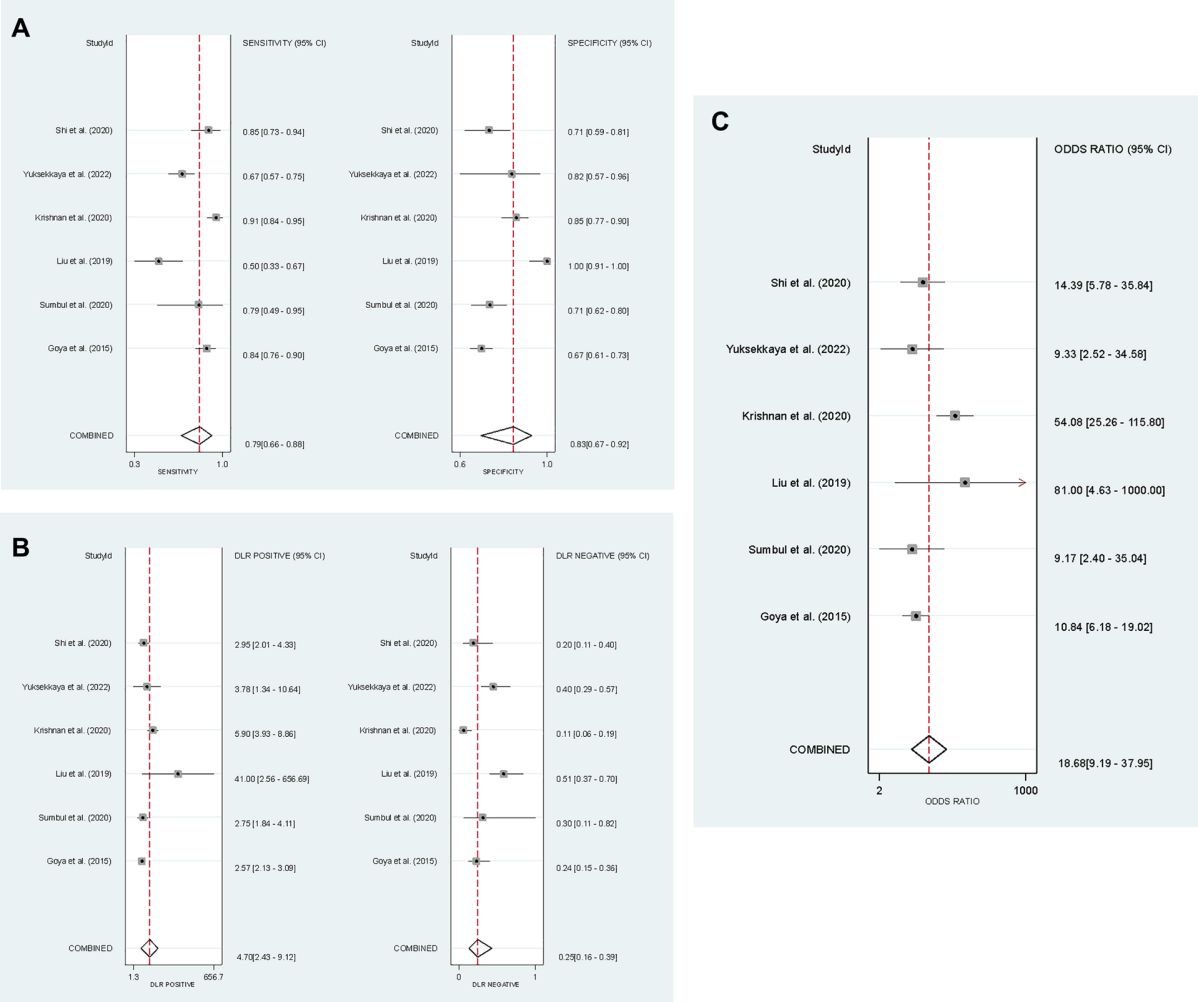
Results of the diagnostic test accuracy (DTA) meta-analysis. The forest plot in A illustrates the pooled sensitivity and specificity for the best cut-off point of cortical stiffness used to differentiate early disease stages (−1 + 0) vs. advanced stages (1 + 2 + 3 + 4 + 5). Plot B displays the pooled positive likelihood ratio (PLR) and negative likelihood ratio (NLR) for the same diagnostic threshold. Finally, Plot C shows the pooled diagnostic odds ratio (DOR) for differentiating these respective DKD groups.

**Fig. 4:**
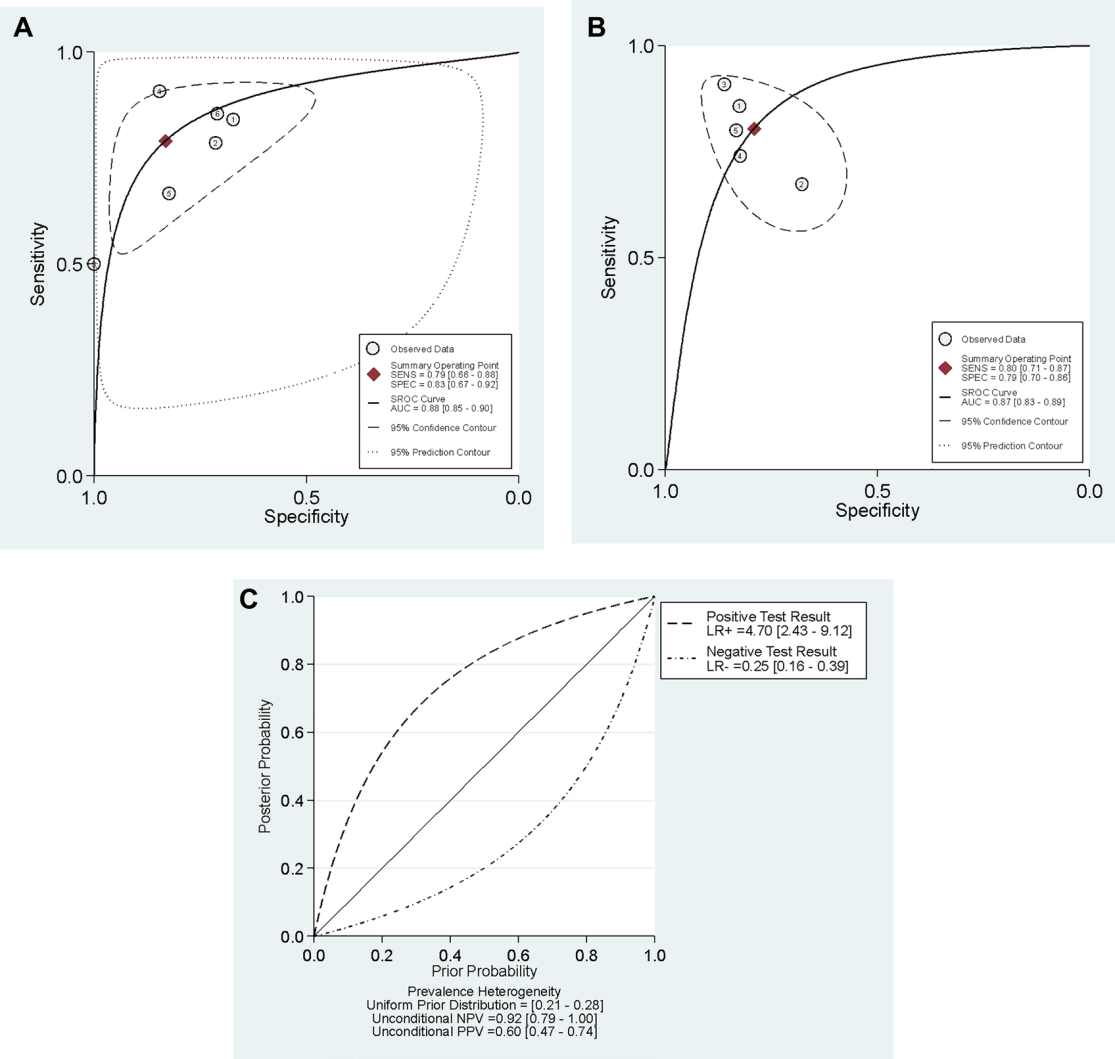
Hierarchical summary receiver operating characteristic (HSROC) curve for cortical stiffness differentiating (−1 + 0) vs. (1 + 2+3 + 4+5) stages in plot A, and (−1 + 0+1 + 2) vs. (3 + 4+5) stages in plot B. The central diamond symbol on the plot shows the summary operating point and the the dashed line surrounding this summary point represents the 95% confidence intervals. The Fagan nomogram in plot C displays the posterior probability values across a range of different prior probabilities for both a negative and a positive USE result. This illustrates the clinical utility of the test by showing how a positive or negative stiffness measurement changes the probability of having advanced DKD in clinical practice.

Six studies^5,6,10,27,29,30^ were included, with 649 participants without DKD and 456 DKD patients. The meta-analysis revealed that USE stiffness measurements exhibited a pooled sensitivity of 79% (CI = 66–88%) and a specificity of 83% (CI = 67–92%) for detecting DKD. Figure 3 shows the detailed diagnostic parameters (i.e., sensitivity, specificity, PLR, NLR, and DOR). Figure 4A represents the hierarchical summary receiver operating characteristic (HSROC) curve with an AUC of 0.88 (CI = 0.85 to 0.90). We also plotted a Fagan nomogram to calculate the pooled positive predictive value (PPV) and negative predictive value (NPV) based on different DKD prevalences (Fig. 4C). No publication bias was present (Appendix E). The generalized I² in this subheading was 70.2%. A sensitivity analysis excluding the study by Krishnan et al.^5^ reduced the I² to 0%.

### Diagnostic value of USE for differentiating (−1 + 0+1 + 2) vs. (3 + 4+5) stages

Five studies^6,11,14,29,33^ were included, with 254 micro- or macroalbuminuria patients and 222 participants without DKD or normoalbuminuria DKD patients. Our analysis revealed that USE stiffness measurements exhibited a pooled sensitivity of 80% (CI = 71–87%) and a specificity of 79% (CI = 70–86%) for the differentiation of the mentioned groups. The generalized I² for this analysis was 0.03%. Comprehensive diagnostic performance parameters are displayed in Appendix F. Also, the HSROC curve in Fig. 4B revealed an AUC of 0.87 (CI = 0.83 to 0.89). No publication bias was present (Appendix E).

### Certainty of evidence

The results of this study are summarized in Table 3 based on the GRADE method for policy-making and clinical context implementation. The results were highly certain in strength across all subheadings of results.

**Table 3:** Certainty of evidence based on grading of Recommendations, Assessment, Development, and evaluations (GRADE) assessment tool.

Outcome	Number of studies	Number of patients	Diagnostic results	Risk of bias	Applicability and indirectness	Inconsistency	Imprecision	Publication bias	Strength of effect size	Certainty
Group comparison based on cortical stiffness	18	2758	Figure 2; Table 2, Appendix C	Moderate risk	Moderate risk	Consistent	Precise	Low risk	High Strength	High ⊕⊕⊕⊕
Diagnostic value of cortical stiffness for differentiating (−1+0) vs. (1+2+3+4+5) stages	6	1105	Figs. 3 and 4	Moderate risk	Moderate risk	Consistent	Precise	Low risk	High Strength	High ⊕⊕⊕⊕
Diagnostic value of cortical stiffness for differentiating (−1+0+1+2) vs. (3+4+5) stages	5	476	Figs. 3 and 4	Moderate risk	Moderate risk	Consistent	Precise	Low risk	High Strength	High ⊕⊕⊕⊕

## Discussion

With its hidden onset, DKD is a progressive, irreversible disease characterized by intricate, long-term pathological changes to renal tissue. Early diagnosis of diabetes-induced nephropathy can greatly impact patients’ long-term prognosis, as timely, proper treatments can remarkably slow the rate of disease advancement^4^. Elastography is considered highly promising for the early identification of DKD and for differentiating between its various stages, hence addressing the limitations of conventional diagnostic procedures^10,11^.

Conventional renal ultrasonography reveals morphological findings, such as changes in cortical and medullary thickness, that may indicate renal parenchymal involvement^7,18^. Five studies^4,7,14,18,31^ included in our research compared cortical thickness between normal healthy participants, diabetic patients without DKD, and different stages of the disease. According to the results published by these studies, progression from early DKD to advanced DKD stages was associated with a reduction in cortical thickness. In contrast, normal healthy controls were found to have lower cortical thickness when compared to diabetic patients without nephropathy or even early-stage DKD patients. This stems from the hyper-filtration state in the asymptomatic phase of the disease, where the earliest pathological changes first occur, which causes cortical parenchymal thickness to increase. Subsequent advancements in stages later in the course of the disease lead to a reduction in cortical thickness^34^. As for medullary thickness, two studies^14,31^ measured its values among healthy controls and different DKD stages, and both found no substantial changes between normoalbuminuria, microalbuminuria, and macroalbuminuria groups and the control group regarding this parameter. According to these reports, neither cortical nor medullary thickness can be used to stage or monitor DKD.

Besides conventional ultrasonography and elastography, there were four studies in our systematic review that evaluated Doppler ultrasound^5–7,30^, investigating the resistive index values in different stages of DKD as well as healthy participants. It is proposed that as DKD progresses, some changes occur in tissue characteristics, which, in turn, may lead to hemodynamic alterations that can be detected^30^. However, the included studies demonstrated that resistive index changes less dramatically than stiffness with DKD progression, suggesting it may be less sensitive for detecting subtle disease advancement.

Our study evaluated whether USE can be used to distinguish between different stages of DKD. We evaluated USE examination results across 5 groups: healthy control patients, DM patients without DKD, DKD patients with normoalbuminuria, DKD patients with microalbuminuria, and DKD patients with macroalbuminuria. We found a trend whereby the stiffness value consistently increases with the progression of the disease, suggesting that renal stiffness measurement can be a valuable tool for assessing kidney injury in patients with DM. The substantial overlap in confidence intervals of cortical stiffness values across albuminuria phenotypes, as illustrated in the forest plots, raises important considerations regarding the clinical application and discriminative utility of stiffness measurements. While mean stiffness values demonstrate progressive increase with advancing albuminuria severity, the breadth of confidence intervals means that individual patients cannot be classified with perfect accuracy into albuminuria categories using stiffness measurements alone. This overlap is not an artifact of measurement error, but rather reflects genuine biological heterogeneity in DKD phenotypes. Albuminuria phenotypes exist on a continuum rather than as discrete categorical entities, and individual patients exhibit substantial variation in the relationship between structural kidney changes and proteinuria levels. From a clinical perspective, this heterogeneity and overlap do not negate the utility of stiffness measurements. Rather, stiffness measurements should be interpreted as complementary biomarkers that provide additional prognostic and staging information within the context of comprehensive clinical assessment including albuminuria status, eGFR, and other markers of kidney disease progression. Methodologically, when comparing groups, the percentage change is less susceptible to bias compared to MD and SMD. This is due to the fact that division, the operation employed in calculating percentage change, is less affected by intra-observer variability than subtraction, which is utilized in MD and SMD. The large values in Table 2 further support the strong association between renal stiffness and the severity of DKD. All of the pairwise percentage change values indicated a “large difference” in their interpretation magnitude zones, showing the potential of USE for accurately staging renal function in DM patients. It is noteworthy that the cortical stiffness in DM patients without DKD demonstrated considerable variation (range: 5.5–77.9 kPa). The decision against quantitative data pooling for this subgroup was based on the limited sample size and study numbers and the wide CI related to this group. Considering our promising result for renal ultrasound elastography, future studies should investigate the potential of other elastography methods, including magnetic resonance elastography (MRE), to enhance the evaluation of renal fibrosis more reliably^35^.

Our results for USE performance in detecting DKD patients from other participants (−1 + 0 stages vs. 1 + 2+3 + 4+5 stages) indicate the interpretation zone of “good” for AUC^21^ and “moderate informativeness” for positive and negative likelihood ratios^20^. These interpretation zones further confirm the previous findings of our study. Certain studies reported the stiffness threshold value for the identification of DKD using USE. Sumbul et al.^27^ and Yuksekkaya et al.^29^ both reported the best cut point value of 9.2 kPa for the differentiation of DKD patients from the control group. Shi et al.^30^ reported a higher best cut point value of 31.7 kPa in this regard. Also, for differentiating (−1 + 0+1 + 2) vs. (3 + 4+5) stages, interpretation zones were “good” for AUC and “moderate informativeness” for positive and negative likelihood ratios. Moreover, clinical centers can determine their own post-test probability of having DKD based on the prevalence of DM patients based on the Fagan nomogram in Fig. 4C. For example, a meta-analysis^35^ suggested 25% (CI = 21% to 28%) for the prevalence of DKD in DM patients in the United States, resulting in an approximate PPV of 60% and NPV of 95% for cortical stiffness examination results. The results of our meta-analysis indicate great reliability, as evidenced by the low heterogeneity observed. The diagnostic threshold distinguishing (− 1 + 0+1 + 2) from (3 + 4+5) stages displayed low heterogeneity (I² = 0.03%). Moreover, in a section characterized by high heterogeneity (I² = 70.2%), a targeted sensitivity analysis effectively identified the sources of variability and diminished the I² value to 0%, hence affirming the stability and validity of the final pooled diagnostic estimations.

The interpretation of our results should be considered in conjunction with the QUADAS-2 and GRADE evaluations, which together highlight the strength of our findings. The GRADE evaluation was consistently high across all subheading of results, influenced by a substantial number of included studies, low heterogeneity, absence of publication bias, and the good diagnostic efficacy of USE. The strong evidence supports that our findings may influence policy-making related to DKD staging. Moreover, the QUADAS-2 tool demonstrated a generally low risk of bias in most of the research domains. The primary methodological limitation noted was the exclusion of patients with prevalent diabetes-related comorbidities, such as hypertension and cirrhosis. This method may have reduced variability, but it introduces a potential selection bias that constrains the external validity of reported results.

Despite USE showing promising results for differentiation of DKD stages, this study has several limitations: (1) Contrary to our protocol, a planned meta-analysis comparing macroalbuminuria (Stages 4–5) to other patients could not be performed due to the absence of reported 2 × 2 diagnostic data. (2) The low number of primary studies that included and assessed diabetic individuals without DKD prevented evaluation of the cortical stiffness for this group. Consequently, although our findings strongly support the staging efficacy of USE in the already diagnosed diabetes cohort, the conclusions concerning the earliest phases necessitate validation by future studies. (3) In this study, we evaluated Emean as the main elastography parameter used in clinical settings; however, other elastography parameters, including Emax and Emin, should also be assessed in future studies. (4) Low inter-observer agreement is an issue in USE examination, as the technique relies on operator expertise^26^. Also, USE examination might be affected by a variety of factors, such as changes in ROI depth, geometry of acquisition, thickness of subcutaneous fat, and body mass index (BMI) of patients^6,14,29^.

## Conclusion

To judge diabetic kidney disease in type 2 DM, USE can be used to measure renal cortical stiffness. As the renal damage progresses, stiffness increases, which is important for the accurate staging of DKD. Future efforts to standardize examination protocols are essential to translate this promising technique into a reliable clinical tool that can enhance patient stratification and management across different healthcare settings.

## Supplementary Information

Below is the link to the electronic supplementary material.

Supplementary Material 1

Supplementary Material 2

Supplementary Material 3

Supplementary Material 4

Supplementary Material 5

Supplementary Material 6

## Data Availability

The data supporting this article can be obtained from the corresponding author upon request.

## Abbreviations

DKD: diabetic kidney disease
USE: ultrasound elastography
DM: diabetes mellitus
SWE: shear wave elastography
DTA: diagnostic test accuracy
MD: mean differences
SMD: standardized mean differences
AUC: area under curve
