# Supplementary material for: To explore the performance of ultrasound elastography in staging diabetic kidney disease: a systematic review and meta-analysis
Source: Sci Rep. 2026 Feb 6;16:7542. doi: 10.1038/s41598-026-39278-w (PMC12932849; doi:10.1038/s41598-026-39278-w)
Supplement: Supplementary file 1 — Supplementary Material 1 [file 41598_2026_39278_MOESM1_ESM.docx]

| Searched database | Search syntax |
| --- | --- |
| PubMed, Web of Science, Embase, and Cochrane library | elastography OR elastogram OR acoustography OR "Acoustic Radiation Force Impulse" OR Sonoelastography OR "shear wave" OR SWV OR kPa OR m/s) AND (diabetes OR diabetic OR DM) AND (renal OR kidney OR nephropathy OR glomerular OR glomerulosclerosis) |

All databases were searched through Ovid simultaneously.
