# Supplementary figures and images for: To explore the performance of ultrasound elastography in staging diabetic kidney disease: a systematic review and meta-analysis
Source: Sci Rep. 2026 Feb 6;16:7542. doi: 10.1038/s41598-026-39278-w (PMC12932849; doi:10.1038/s41598-026-39278-w)

Risk of bias assessment based on QUADAS-2.


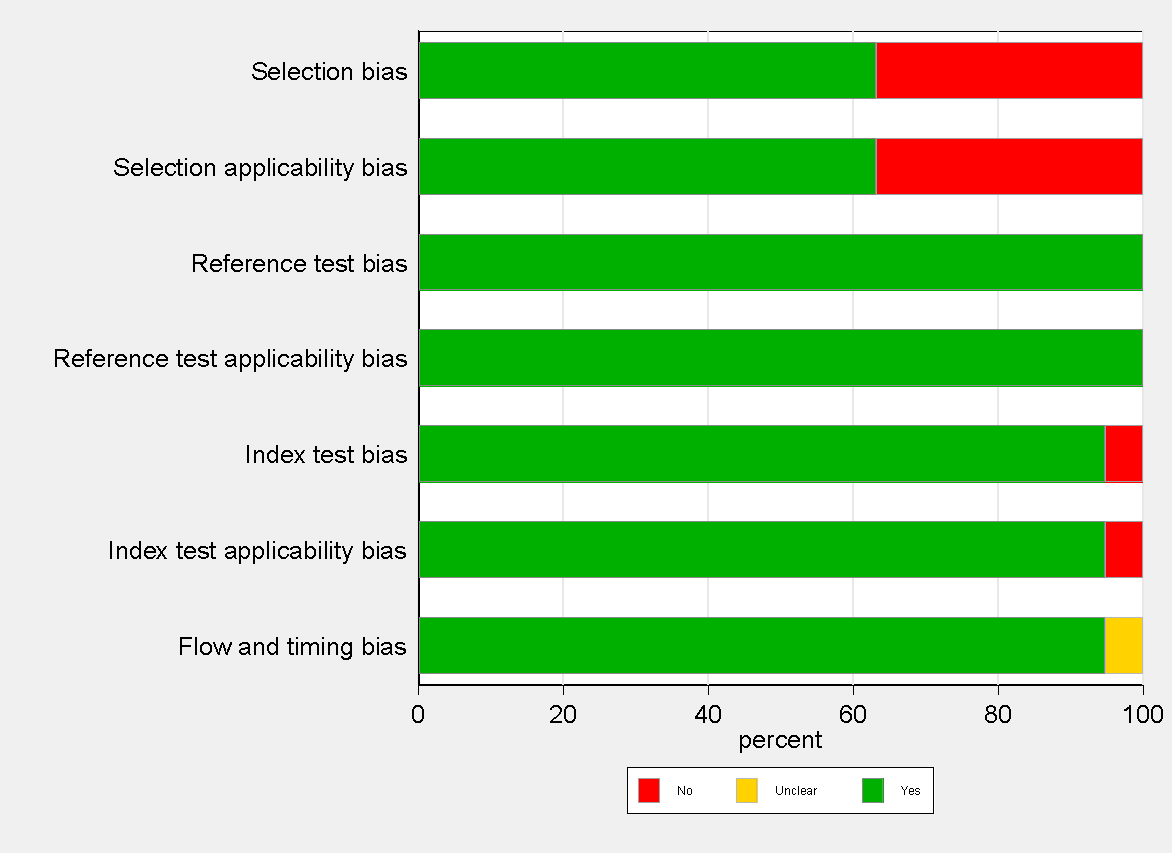

Supplement: Supplementary file 2 — Supplementary Material 2 [file 41598_2026_39278_MOESM2_ESM.docx]

Differentiating (-1+0) vs. (1+2+3+4+5) stages:


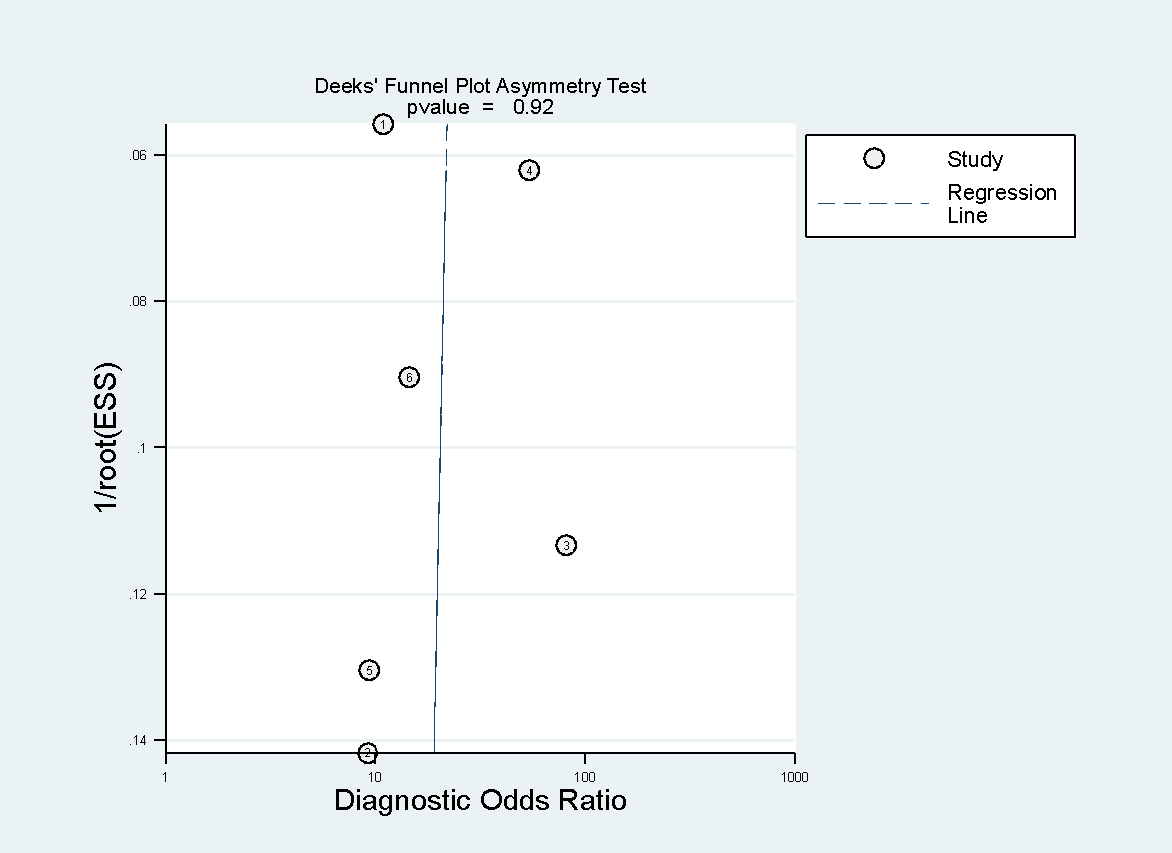


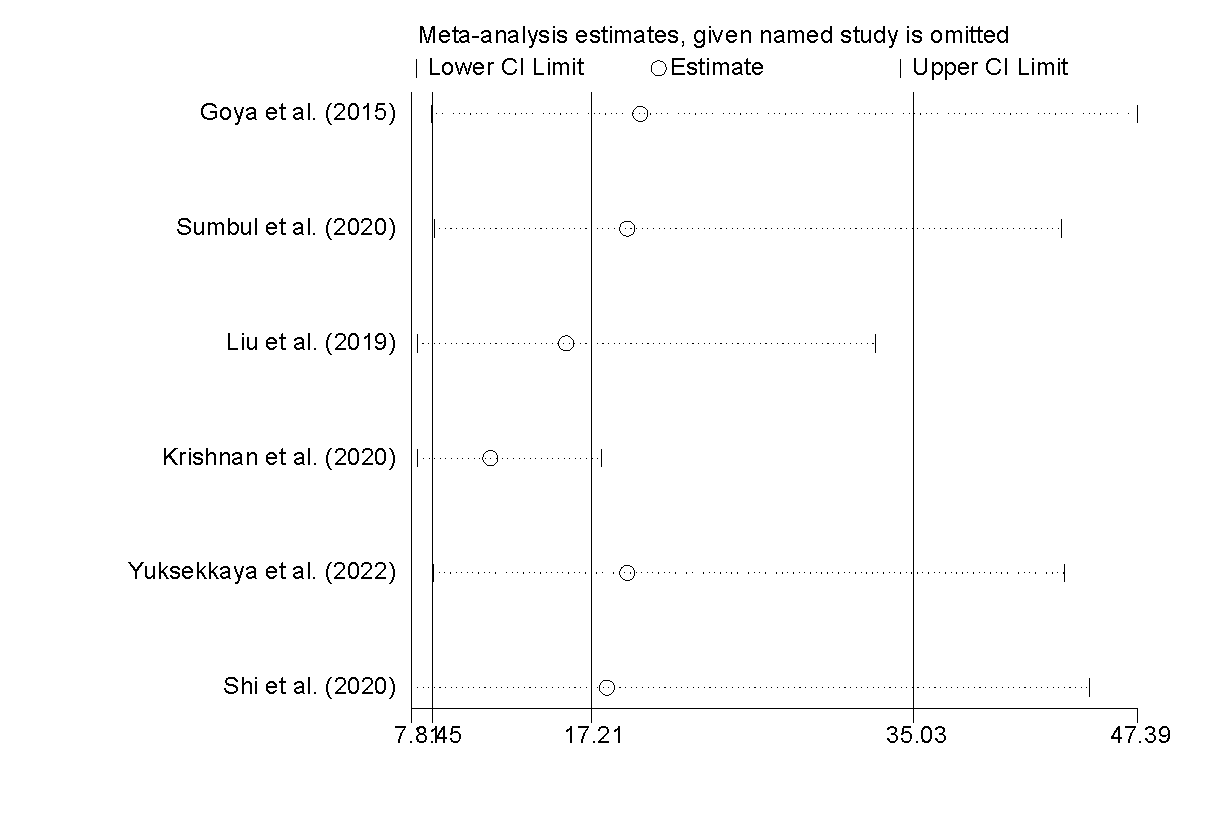


=====================================================================================

Differentiating (-1+0+1+2) vs. (3+4+5) stages


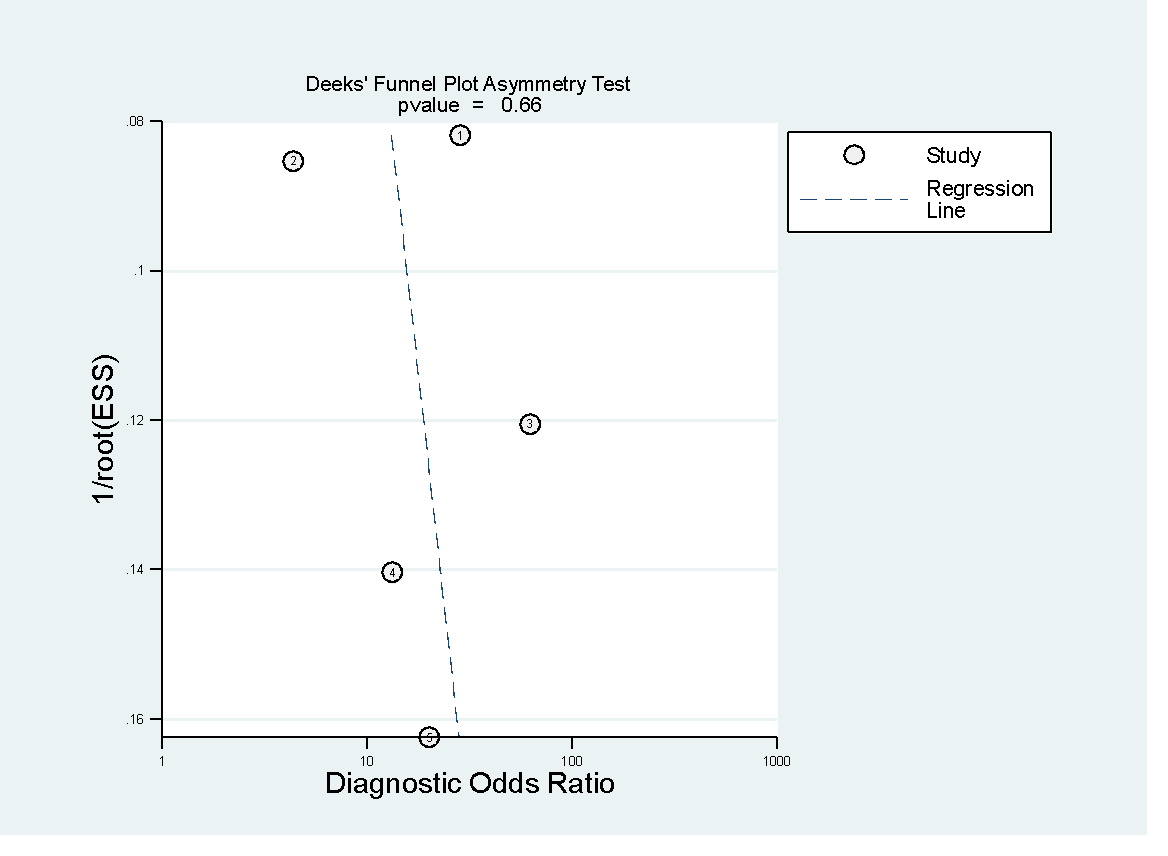


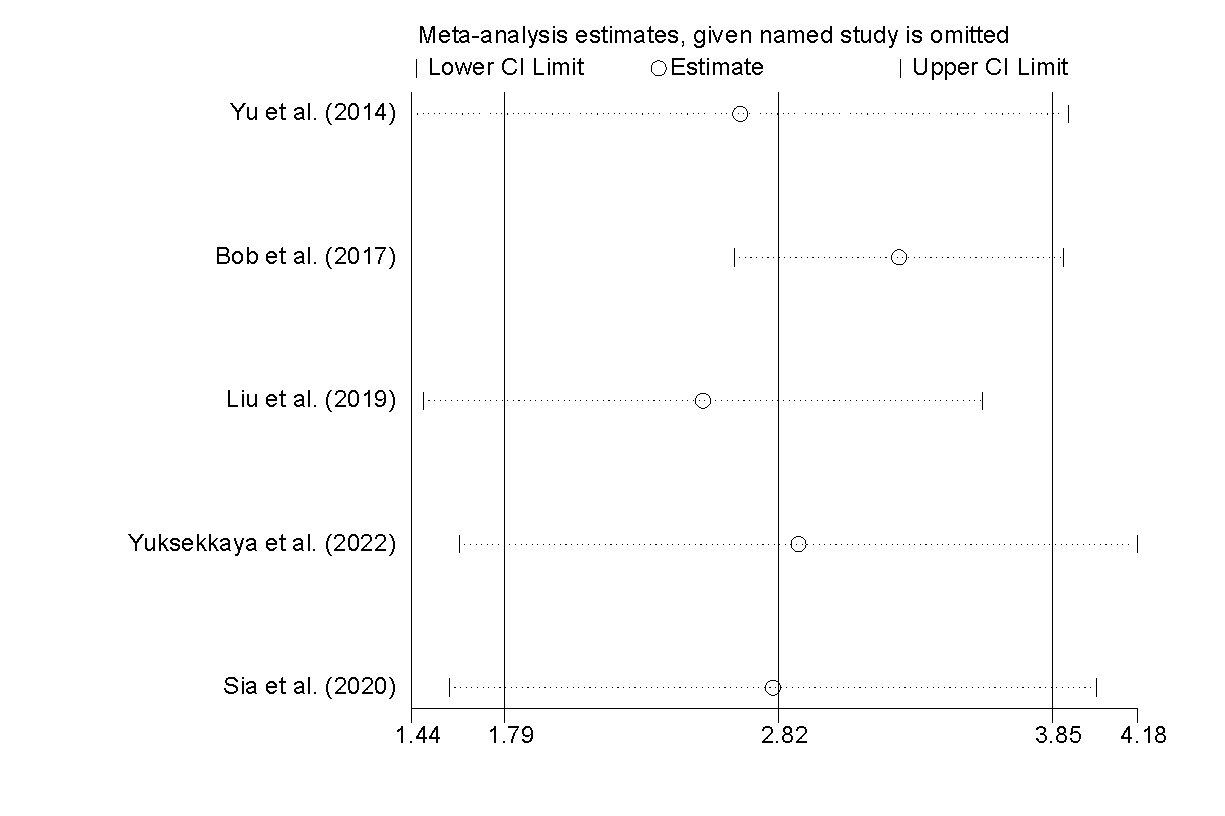

Supplement: Supplementary file 4 — Supplementary Material 4 [file 41598_2026_39278_MOESM4_ESM.docx]
