# Supplementary material for: To explore the performance of ultrasound elastography in staging diabetic kidney disease: a systematic review and meta-analysis
Source: Sci Rep. 2026 Feb 6;16:7542. doi: 10.1038/s41598-026-39278-w (PMC12932849; doi:10.1038/s41598-026-39278-w)

1. Sensitivity and specificity for the best cut point of cortical stiffness for differentiating stages of (-1+0+1+2) vs. (3+4+5).


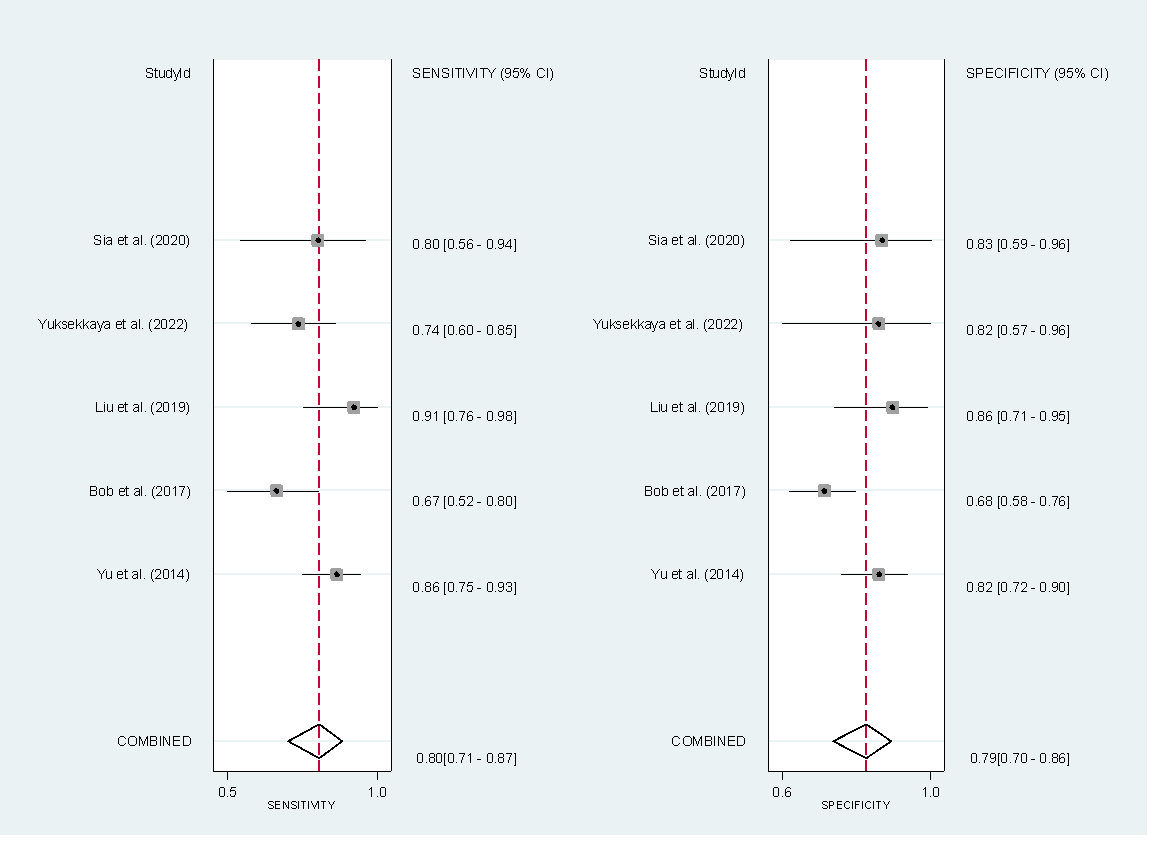


B) Positive and negative likelihood ratios for the best cut point of cortical stiffness for differentiating the same mentioned groups.


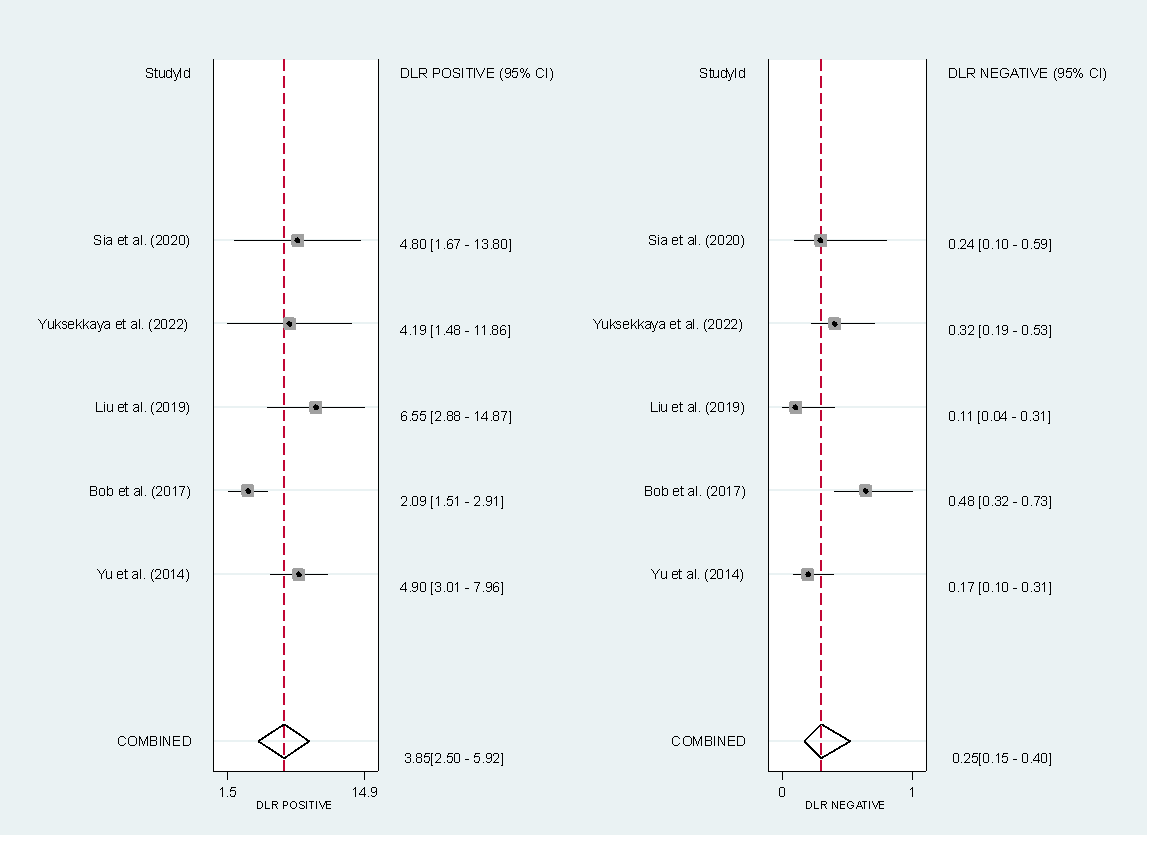


C) Diagnostic odds ratio for best cut point of cortical stiffness for differentiating the same mentioned groups.


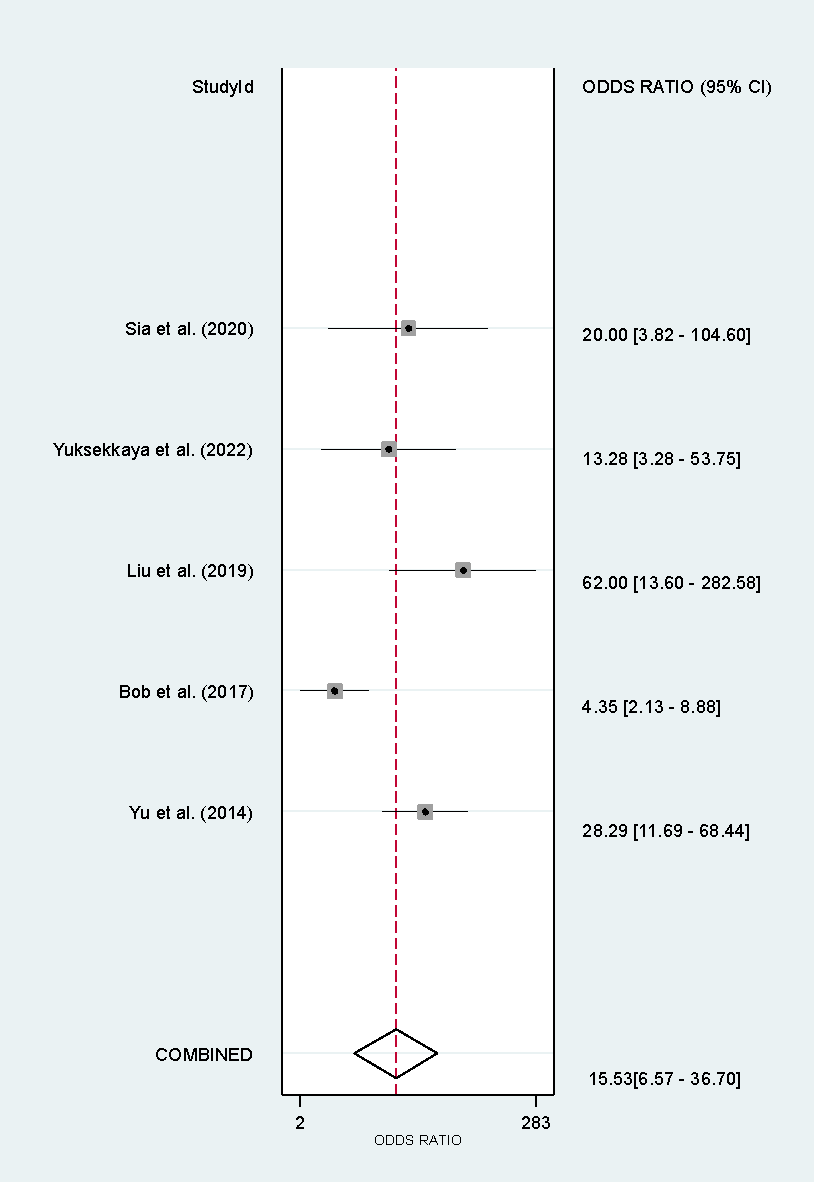

Supplement: Supplementary file 3 — Supplementary Material 3 [file 41598_2026_39278_MOESM3_ESM.docx]
