# Supplementary material for: To explore the performance of ultrasound elastography in staging diabetic kidney disease: a systematic review and meta-analysis
Source: Sci Rep. 2026 Feb 6;16:7542. doi: 10.1038/s41598-026-39278-w (PMC12932849; doi:10.1038/s41598-026-39278-w)
Supplement: Supplementary file 5 — Supplementary Material 5 [file 41598_2026_39278_MOESM5_ESM.docx]

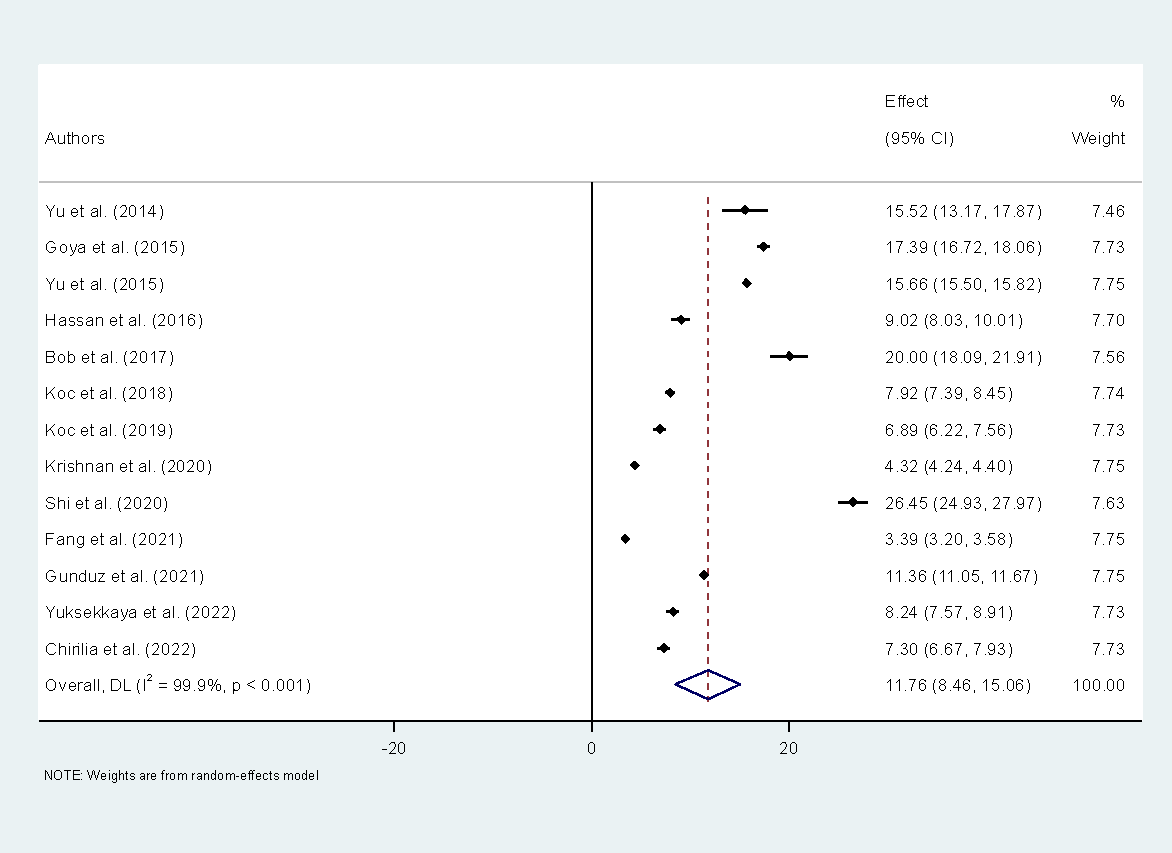


Healthy control patients

=====================================================================================

DM patients without DKD

=====================================================================================


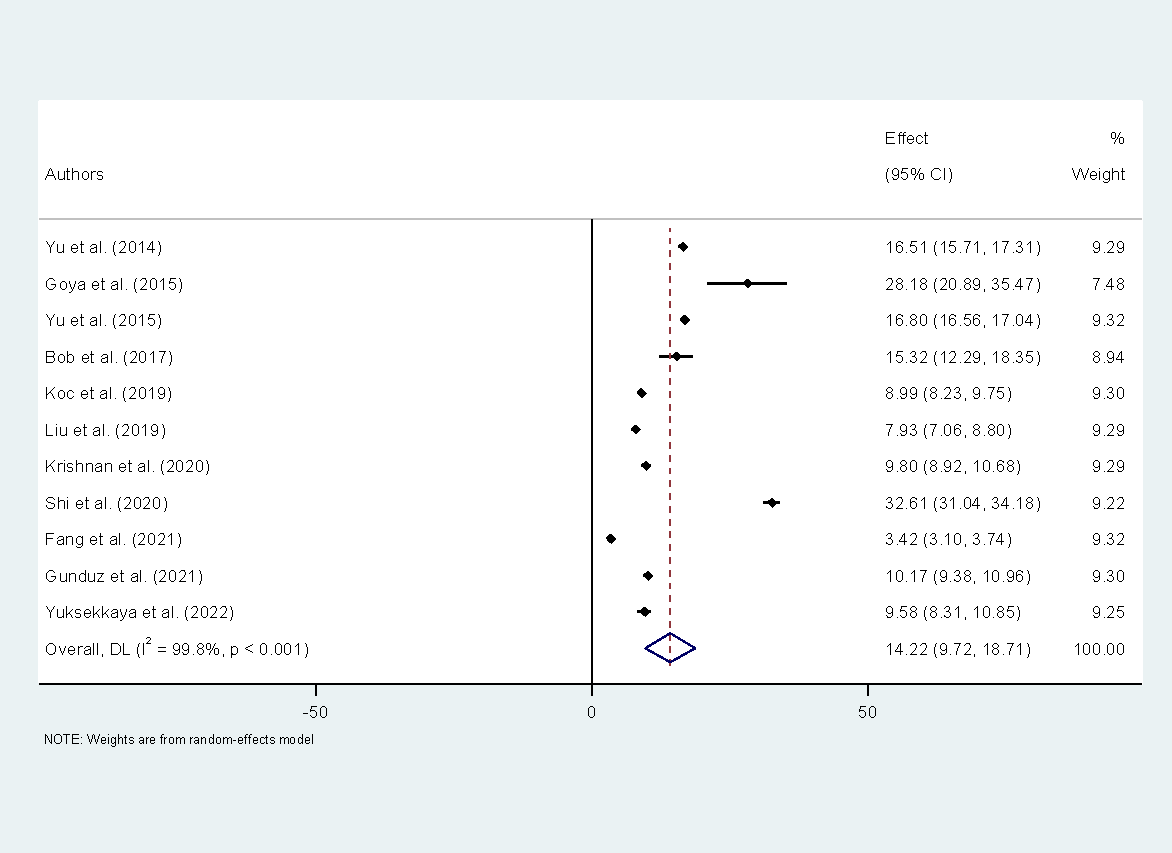


DKD patients with normoalbuminuria

=====================================================================================


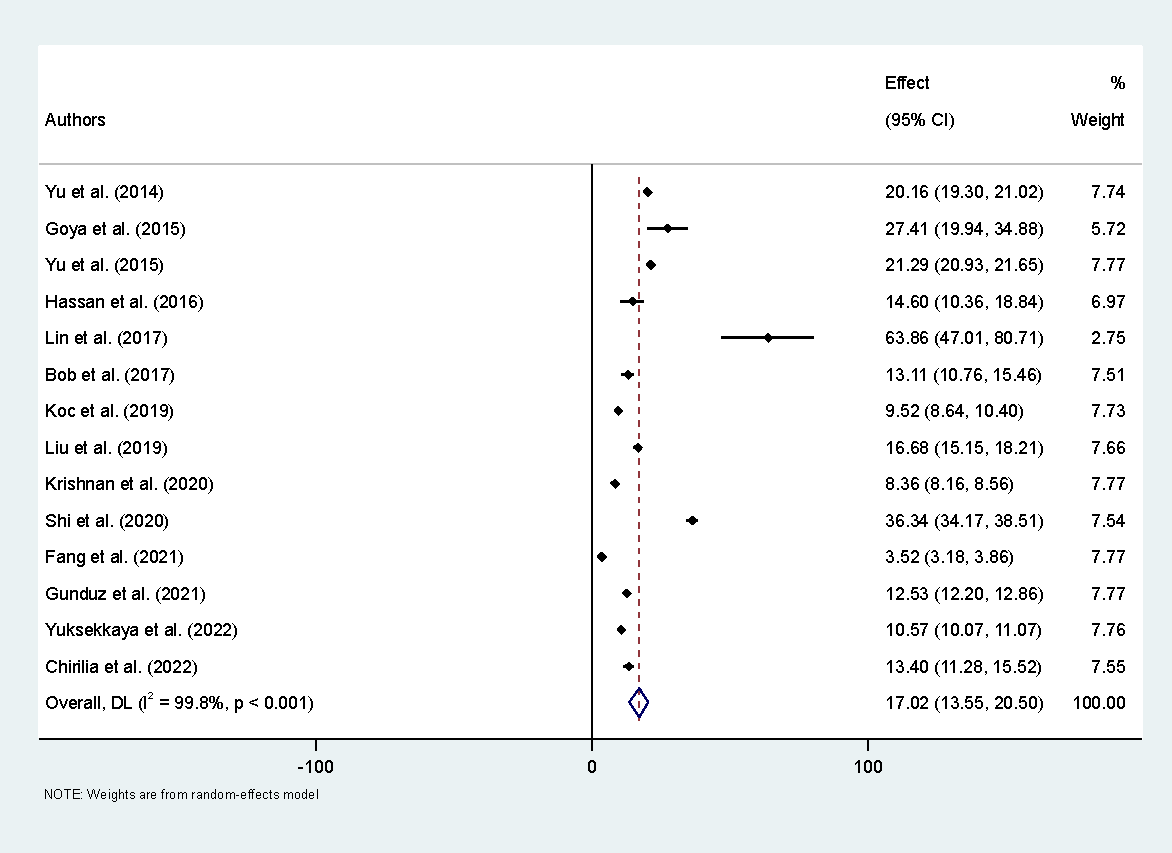


DKD patients with microalbuminuria

=====================================================================================


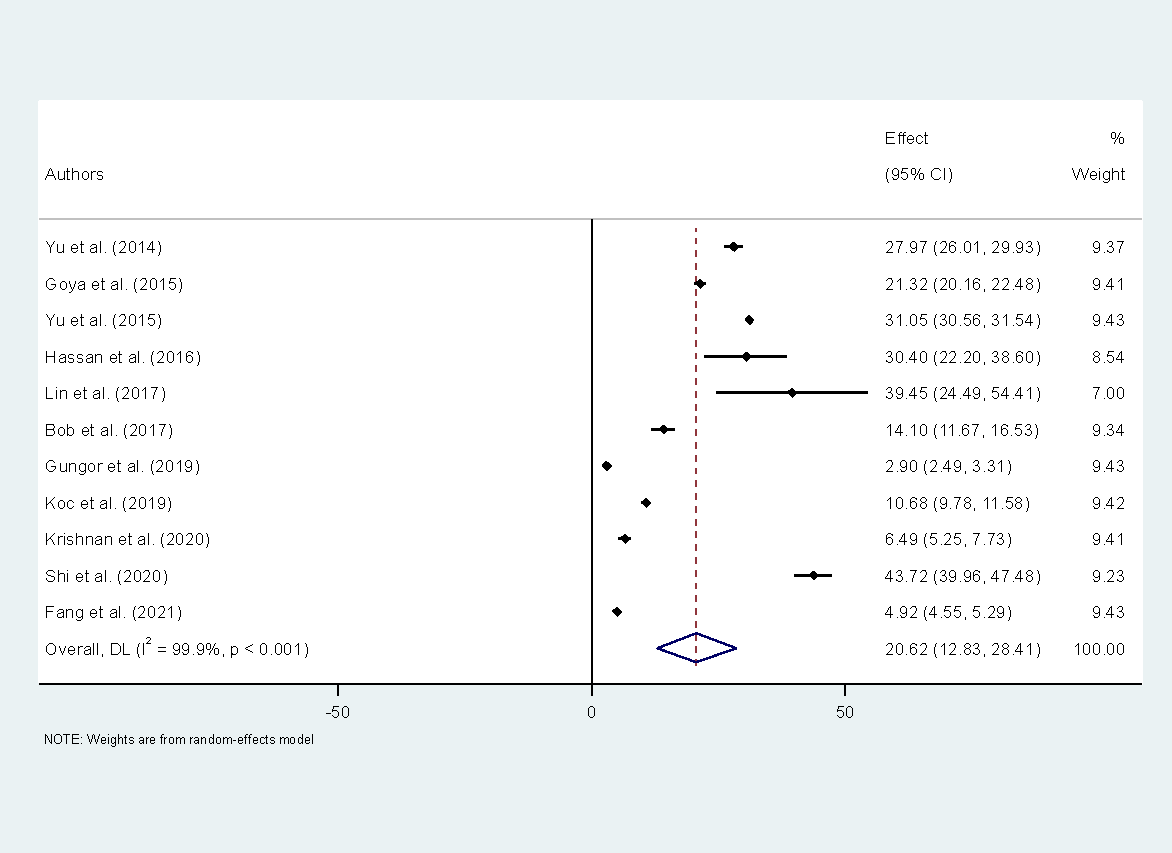


DKD patients with macroalbuminuria

===================================================================
