# Supplementary material for: To explore the performance of ultrasound elastography in staging diabetic kidney disease: a systematic review and meta-analysis
Source: Sci Rep. 2026 Feb 6;16:7542. doi: 10.1038/s41598-026-39278-w (PMC12932849; doi:10.1038/s41598-026-39278-w)
Supplement: Supplementary file 6 — Supplementary Material 6 [file 41598_2026_39278_MOESM6_ESM.pdf]

Diagnostic performance of ultra-sound elastography for detection of diabetic nephropathy: A systematic review and meta-analysis.

1. Review question:
To determine diagnostic performance of ultrasound elastography for detection of diabetic nephropathy

2. Searches:
Reviewers A.Mohebbi. and S.M. and M.H.E developed their search syntax independently. Additionally, EMTREE and MeSH keywords were checked. After discussing the differences, they merged their strategies. If the discussion did not lead to a conclusion, a fourth reviewer (A.Mohammadi.) was involved. A finalized search syntax will be conducted on September 29, 2023, and all eligible studies will be included. We will search PubMed, Web of Science, Embase, and Cochrane Library databases. No language restrictions will be applied. Additionally, conference abstracts will be included. Furthermore, the reference lists of the relevant papers will be reviewed to ensure no publications are missed. To perform screening, all searched studies will be imported into EndNote software. The duplicate studies will then be removed.

(elastography OR elastogram OR acoustography OR "Acoustic Radiation Force Impulse" OR Sonoelastography OR "shear wave" OR SWV OR kPa OR m/s) AND (diabetes OR diabetic OR DM) AND (renal OR kidney OR nephropathy OR glomerular OR glomerulosclerosis)

3. Types of study to be included:
Inclusion criteria will include, case-control, cohort, cross-sectional, and clinical trials with more than five patients.

4. Condition or domain being studied:
Long-term diabetics suffer from kidney disease, which is a life-threatening issue. This causes end-stage kidney disease (ESRD) and places a heavy burden on healthcare systems. Before clinical nephropathy occurs, early changes in cortical cells take place in the course of the disease. A renal biopsy is still the gold standard for evaluating these changes, but it is an invasive procedure that cannot be used for early diagnosis or serial monitoring of diabetic kidney disease (DKD). Unlike invasive methods of assessing renal stiffness, ultrasonography (US) is a noninvasive alternative that is becoming increasingly popular. US-based imaging method is FDA-approved and highly diagnostic in detecting liver diseases. In addition, recent studies have demonstrated that it can be used to evaluate renal fibrosis and stage chronic nephropathy.

5. Intervention(s), exposure(s):
Shear wave elastography (SWE) is a noninvasive imaging technique that measures tissue stiffness by generating and tracking shear waves. By using focused acoustic energy, shear waves are generated, which propagate perpendicular to the acoustic beam direction. The ultrasonic scanner measures the displacement of the tissue as the shear waves pass through it, and calculates the peak displacement and recovery times. Measurements of tissue stiffness are expressed in meters per second (m/s) or kilopascals (kPa). Fibrotic tissues typically have a higher

shear wave velocity and hardness than normal tissues. SWE can be used to detect and quantify tissue stiffness in a variety of organs, including the liver, kidneys, and pancreas.

6. Comparator(s)/control:
The diagnostic performance of shear wave elastography will be determined in healthy participants as well as diabetic patients with and without nephropathy.

7. Main outcome:
To determine the diagnostic performance of shear wave elastography parameters in differentiation of healthy individuals as well as diabetics with and without nephropathy.

8. Additional outcome(s):
A) To directly compare SWE findings (e.g., velocity, pressure, resistive index, echogenicity, cortical thickness, medullary thickness) for each of participant groups and calculate the effect size (e.g., standardized mean difference (SMD)) if enough data are reported in the studies.
B) To evaluate the subclassification based on QUADAS-2 findings as well as number of radiologists used to perform the ultrasonography and different grades of nephropathy if enough data are reported.

C) To determine best cut point value for abovementioned parameters for differentiating the groups

D) To perform publication bias evaluation on upper results if enough data are available.

E) To perform sensitivity analysis on upper results if enough data are available.

F) To perform GRADE assessment on upper results if enough data are available.

9. Data extraction (selection and coding):
In order to answer the review question, three reviewers (A.Mohebbi. and S.M. and M.H.E) will independently extract data from the included studies. Next, the data will be entered into an Excel sheet, and the two files will be compared to determine if there are any differences. A fourth reviewer (A.Mohammadi.) will solve disagreements between them if the differences cannot be resolved.

10. Risk of bias assessment:
The bias of the included studies was assessed with Quality Assessment of Diagnostic Accuracy Studies-2 (QUADAS-2) including some additional comments recommended by Cochrane Handbook for Systematic Reviews of Diagnostic Test Accuracy.

11. Strategy for data synthesis:
The meta-analysis will be conducted using STATA version 17.0 and MetaBayes applications. MedCalc version 20 will be used for direct comparison. A random-effects model will be used, as recommended by the Cochrane Handbook for Systematic Reviews of Diagnostic Test Accuracy for diagnostic studies. The $I2 \geq 50\%$ will be considered a high statistical heterogeneity model. 12. Contact details for further information:
Afshin Mohammadi

Afshin.mohdi@gmail.com
Full Professor of Diagnostic and Interventional Radiology. Urmia University of Medical Sciences

13. Organizational affiliation of the review:
Universal Scientific Education and Research Network (USERN), Tehran, Iran.
Students' Scientific Research Center (SSRC), Tehran, Iran.

14. Review team members and their organizational affiliations:

Alisa Mohebbi Universal

Scientific Education and Research Network (USERN), Tehran, Iran.

Saeed Mohammadzadeh

Students' Scientific Research Center (SSRC), Tehran, Iran.

Mohammadhossein Esalmi

Universal Scientific Education and Research Network (USERN), Tehran, Iran.

15. Anticipated or actual start date:
29 September 2023

16. Anticipated completion date:
1 December 2023

17. Funding sources or sponsors:
None

18. Conflicts of interests:
None
